# Integrated multiomics analysis and machine learning refine neutrophil extracellular trap-related molecular subtypes and prognostic models for acute myeloid leukemia

**DOI:** 10.3389/fimmu.2025.1558496

**Published:** 2025-02-24

**Authors:** Fangmin Zhong, Fangyi Yao, Zihao Wang, Jing Liu, Bo Huang, Xiaozhong Wang

**Affiliations:** Jiangxi Province Key Laboratory of Immunology and Inflammation, Jiangxi Provincial Clinical Research Center for Laboratory Medicine, Department of Clinical Laboratory, The Second Affiliated Hospital, Jiangxi Medical College, Nanchang University, Nanchang, Jiangxi, China

**Keywords:** acute myeloid leukemia, neutrophil extracellular traps, molecular subtypes, machine learning, prognosis, personalized medicine

## Abstract

**Background:**

Neutrophil extracellular traps (NETs) play pivotal roles in various pathological processes. The formation of NETs is impaired in acute myeloid leukemia (AML), which can result in immunodeficiency and increased susceptibility to infection.

**Methods:**

The gene set variation analysis (GSVA) algorithm was employed for the calculation of NET score, while the consensus clustering algorithm was utilized to identify molecular subtypes. Weighted gene coexpression network analysis (WGCNA) revealed potential genes and biological pathways associated with NETs, and a total of 10 machine learning algorithms were applied to construct the optimal prognostic model.

**Results:**

Through the analysis of multiomics data, we identified two molecular subtypes with high and low NET scores. The low-NET score subgroup exhibited increased infiltration of immune effector cells. Conversely, the high-NET score subtype presented an abundance of monocytes and M2 macrophages, accompanied by elevated expression levels of immune checkpoint genes. These findings suggest that a pronounced immunosuppressive effect is associated with a significantly worse prognosis for this subtype. The optimal risk score model was selected by employing the C-index as the criterion on the basis of training 10 machine learning algorithms on 9 multicenter AML cohorts. Survival analysis confirmed that patients with high-risk scores had considerably poorer prognoses than those with lower scores. Receiver operating characteristic (ROC) curve and Cox regression analyses further validated the strong independent prognostic value of the risk score model. The nomogram, which was constructed by integrating the risk score model and clinicopathological factors, demonstrated high accuracy in predicting the overall survival of AML patients. Moreover, patients with refractory or chemotherapy-unresponsive AML had significantly higher risk scores. By analyzing drug therapy data from *in vitro* AML cells, we identified a subset of drugs that demonstrated increased sensitivity in the high-risk score group. Additionally, patients with a high risk score were also predicted to exhibit a favorable response to anti-PD-1 therapy, suggesting that these individuals may derive greater benefits from immunotherapy.

**Conclusion:**

The NET-related signature, derived from a combination of diverse machine learning algorithms, has promising potential as a valuable tool for prognostic prediction, preventive measures, and personalized medicine in patients with AML.

## Introduction

Acute myeloid leukemia (AML) is characterized by impaired differentiation and uncontrolled proliferation of malignant hematopoietic stem/progenitor cells, resulting in the suppression of the normal hematopoietic lineage and significant impairment of blood cell production and immune function (1). Consequently, AML patients are highly susceptible to infectious complications, both as a consequence of the disease itself and treatment-related factors such as chemotherapy (2, 3). These crucial factors significantly contribute to the unfavorable prognosis of patients with AML, underscoring the importance of investigating relevant mechanisms and developing novel prognostic markers for evaluating patient outcomes.

Neutrophil extracellular traps (NETs) are recently discovered extracellular network structures that are primarily released by neutrophils in response to various stimuli. This structure is composed of DNA, histone proteins, and antibacterial proteins and serves as a crucial innate immune defense mechanism against pathogenic microorganisms such as bacteria, fungi, viruses, and protozoa. The process of neutrophil secretion of NETs, known as NETosis, represents a distinct form of neutrophilic death that differs from apoptosis and necrosis. This process necessitates the activation of neutrophils and the generation of reactive oxygen species through NADPH oxidase.

Currently, there are two classifications of NETs: Suicidal NETosis, where neutrophils release NETs before undergoing membrane disintegration and subsequent cell death; and vital NETosis, where neutrophils remain alive after releasing vesicular forms of NETs (4). The impact of NETosis on tumorigenesis and development is twofold (5). On the one hand, by activating the immune system, NETs can inhibit tumor growth. Additionally, through the release of NETs, neutrophils interact with T cells to lower their activation threshold and directly stimulate antitumor immune effects (6). On the other hand, tumors can induce neutrophil-mediated NETosis and promote tumor metastasis, thereby contributing to cancer progression (7). In patients with AML, immature differentiation of neutrophils and inadequate chromatin concentrations pose challenges in releasing chromatin into the extracellular space to form functional NETs (8). This directly affects AML patients’ ability to combat bacterial infections, which are a significant cause of mortality during AML treatment (9). Several studies have confirmed the impaired release of NETs in AML patients (10, 11); however, the underlying mechanisms remain incompletely understood. Therefore, analyzing the relationships between the expression of genes related to NET formation and prognostic features in AML is crucial.

In this study, we computed the NET score on the basis of NET-related genes (NRGs) and assessed its correlation with prognosis, immune characteristics, and cancer-promoting pathways in patients with AML. Additionally, we identified two distinct molecular subtypes related to NETs that exhibited significant disparities in the tumor microenvironment (TME). We subsequently employed ten machine learning algorithms to construct an optimal prognostic risk score model via NRG expression. The accuracy of our model’s prognostic predictions was validated across nine AML cohorts. Finally, we investigated differences in sensitivity to different chemotherapy agents and immunotherapy responsiveness between the high-risk and low-risk groups while also validating the expression of model genes through clinical sample collection.

## Materials and methods

### Data collection and preprocessing

A total of 9 cohorts consisting of AML samples were utilized in this study, encompassing a total of 2061 AML samples whose clinical information was available. These cohorts comprised 8 Gene Expression Omnibus (GEO) datasets, namely, GSE10358-GPL570, GSE12417-GPL96, GSE12417-GPL570, GSE37642-GPL96, GSE37642-GPL570, GSE71014-GPL10558, GSE14688-GPL570 and Beat AML (12). Additionally, we obtained 173 AML samples from the TCGA-LAML cohort and 337 normal blood samples from the GTEx cohort from the UCSC Xena database (https://xena.ucsc.edu/). To ensure consistency across platforms for GEO cohorts on the GPL570 chip platform, we acquired the original “CEL” file and performed data normalization via the robust multiarray averaging (RMA) method. For other GEO cohorts on different platforms, standardized data files were downloaded. RNA sequencing data from the TCGA-LAML, GTEx, and Beat AML cohorts were converted into transcripts per million (TPM) values. Somatic mutation data and gene copy numbers were retrieved from the TCGA database (https://portal.gdc.cancer.gov/). A total of 69 NRGs were obtained from a previous study (13, 14) (**Supplementary Table S1**).

### Analysis of functional enrichment and evaluation of immune cell infiltration

The software package ‘clusterProfiler’ was utilized for performing Gene Ontology (GO) annotation and Kyoto Encyclopedia of Genes and Genomes (KEGG) enrichment analysis (15). The pathway score was calculated via the gene set variation analysis (GSVA) algorithm to quantify the activity level of each pathway (16). The GSVA algorithm initially ranks the expression levels of all genes within a single sample in descending order, followed by an analysis of the positioning of target gene sets within this ranking. If these genes exhibit high expression levels, they will be ranked higher, indicating elevated activity of the corresponding gene set or pathway. In this study, we assessed the scores of the NET gene set as a representation of NET activity within each sample. To estimate the proportions of 22 infiltrating immune cell types, we employed the CIBERSORT algorithm (17). Additionally, the ESTIMATE algorithm was applied to assess both the immune score and matrix score for the entire sample (18).

### Identification of molecular subtypes

The “ConsensusClusterPlus” package was utilized for unsupervised clustering of AML samples, and 1000 resampling iterations were performed to ensure the robustness of the cluster analysis results.

### Weighted gene coexpression network analysis

WGCNA is a systematic biological approach used to identify highly correlated gene sets and characterize patterns of gene associations across different samples. In our study, we utilized the “WGCNA” package to conduct WGCNA (19). Initially, we calculate an appropriate soft threshold β to ensure the construction of a scale-free network. We subsequently transformed the weighted adjacency matrix into a topological overlap matrix (TOM) and computed dissimilarity (dissTOM). To cluster genes and identify modules, we employed the dynamic tree-cutting method. Ultimately, we identified modules that exhibited the strongest correlation with phenotype for further analysis.

### Development of a prognostic model through the integration of machine learning approaches

We utilized genes from modules associated with NET scores and subtypes identified by WGCNA to construct prognostic models. Initially, we employed univariate Cox regression analysis to screen for genes significantly associated with prognosis (P<0.05) in at least five of the nine AML cohorts while maintaining a consistent hazard ratio (HR) orientation. The TCGA-LAML dataset was designated the analysis cohort, while the remaining datasets served as the validation cohort. By incorporating 10 machine learning algorithms, including CoxBoost, stepwise Cox, Lasso, Ridge, elastic net (Enet), survival support vector machines (survival-SVMs), generalized boosted regression models (GBMs), supervised principal components (SuperPC), partial least Cox (plsRcox) and RSF, we performed 117 combinations of these algorithms in the TCGA-LAML training cohort for variable selection and model construction on the basis of a 10-fold cross-validation framework (20, 21). All the constructed models were evaluated in both the validation and analysis cohorts. For each model, its C-index was calculated in both the training and validation cohorts. We subsequently ranked the predictive performance of each model on the basis of its average C-index within the validation cohort. Finally, a combination of algorithms demonstrating robust performance and clinical translational significance was selected to develop a risk score model capable of predicting AML patient prognosis. In this study, the ridge algorithm was used to construct the risk scoring model.


$$Risk\;score=\;\sum_{1}^{i}\left(Coefi*ExpGenei\right)\;,$$

where i is the model gene, and the regression coefficient and expression value are represented by ‘Coef’ and ‘ExpGene’, respectively (**Supplementary Table S3**). Using an optimal cutoff value, all the AML cohorts were stratified into high- and low-risk score groups for further analysis.

### Forecasting the efficacy of immunotherapy and predicting susceptibility to chemotherapy

The SubMap algorithm (https://cloud.genepattern.org/gp) was employed to predict the response of diverse risk score groups to immunotherapy involving anti-PD-1 and anti-CTLA4. Within the Beat AML cohort, we examined potential therapeutic agents suitable for various risk score groups by analyzing drug sensitivity data pertaining to *in vivo* AML cells.

### Transcriptome sequencing of clinical samples from patients with AML

This research was granted approval by the Ethics Committee at the Second Affiliated Hospital of Nanchang University (No. review. [2018] No. (092)). In accordance with the World Health Organization’s classification of tumors in hematopoietic and lymphoid tissues, we obtained 5 newly diagnosed AML samples that had not undergone any prior treatment, as well as 5 normal samples, following established guidelines. All the samples remained after the participants underwent relevant examinations. The procedures and protocols for sample collection, transcriptome sequencing, and processing were described in detail in our previous publication (22).

### Statistical analysis

R software was utilized for conducting the statistical analysis. The Wilcoxon test was used to assess differences between two groups, whereas the Kruskal−Wallis test was used to compare differences among multiple groups. A significance level of P<0.05 was considered (* P<0.05, ** P<0.01, *** P<0.001).

## Results

### Development of a NET scoring system to investigate the potential associations between NETs and the TME

**Figure 1:**
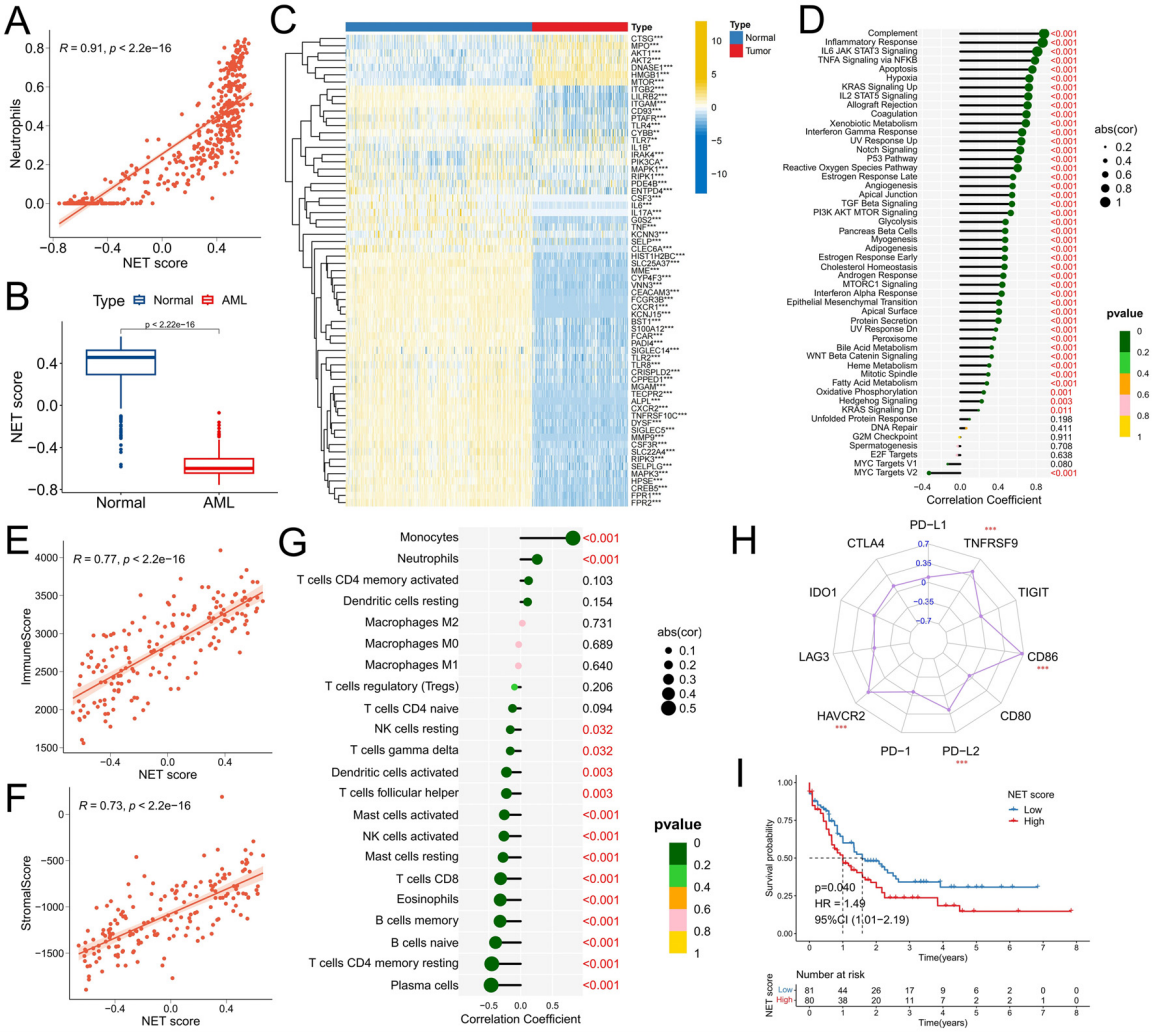
Construction of the NET scoring system and correlation analysis with the TME. **(A)** Association between the NET score and the presence of neutrophils in the TCGA-LAML and GTEx normal blood samples cohorts. **(B)** Comparison of NET scores in samples from patients with AML and healthy individuals. **(C)** Comparison of NRG expression in samples from patients with AML and healthy individuals. **(D-H)** Correlations between the NET score and the activity of cancer signature pathways **(D)**, immune response scores **(E)**, stromal scores **(F)**, infiltration of immune cells **(G)**, and expression of immune checkpoint molecules (The number represents the R-value of the correlation) **(H)** in the TCGA-LAML cohort. **(I)** Survival analysis between different NET score groups in the TCGA-LAML cohort. * P<0.05, ** P<0.01, *** P<0.001.

First, the GSVA algorithm was used to calculate NET scores for both AML samples and normal samples to characterize NET activity. Correlation analysis revealed a significant positive correlation between the NET score and the neutrophil ratio (R=0.91, P<2.2e-16) (**Figure 1A**), confirming the reliability of the NET score calculation method. Notably, compared with normal samples, AML samples presented significantly lower NET scores (**Figure 1B**), which can be attributed to the immature neutrophil differentiation observed in AML samples. Heatmap analysis revealed that most NRG genes were downregulated in AML samples (**Figure 1C**). Furthermore, there was a significant positive correlation between the NET score and the activity of various cancer signature pathways, particularly immune-related pathways such as the complement, IL6-JAK-STAT3 signaling, and inflammatory response pathways (**Figure 1D**), indicating an interconnected relationship between NETs and the immune microenvironment. Subsequent analysis confirmed a significant positive correlation between the NET score and the immune score (R=0.77, P<2.2e-16) as well as the matrix score (R=0.73, P<2.e-16) (**Figures 1E, F**), thus supporting our initial hypothesis. Immune infiltration analysis revealed a significant positive correlation between the NET score and the proportion of infiltrating monocytes and neutrophils, whereas a significant negative correlation was observed with immune effector cells such as plasma cells, CD4+ T cells, B cells, CD8+ T cells, NK cells, mast cells, and dendritic cells (**Figure 1G**). However, high NET scores were associated with increased expression of immune checkpoints such as HAVCR2, PD-L2, CD86, and TNFRSF9 (**Figure 1H**). These findings suggest that the formation and release of NETs may also be involved in the immune evasion of tumor cells (23, 24). Survival analysis demonstrated that patients with high NET scores had significantly worse prognoses (**Figure 1I**).

### The landscape of NRG expression

**Figure 2:**
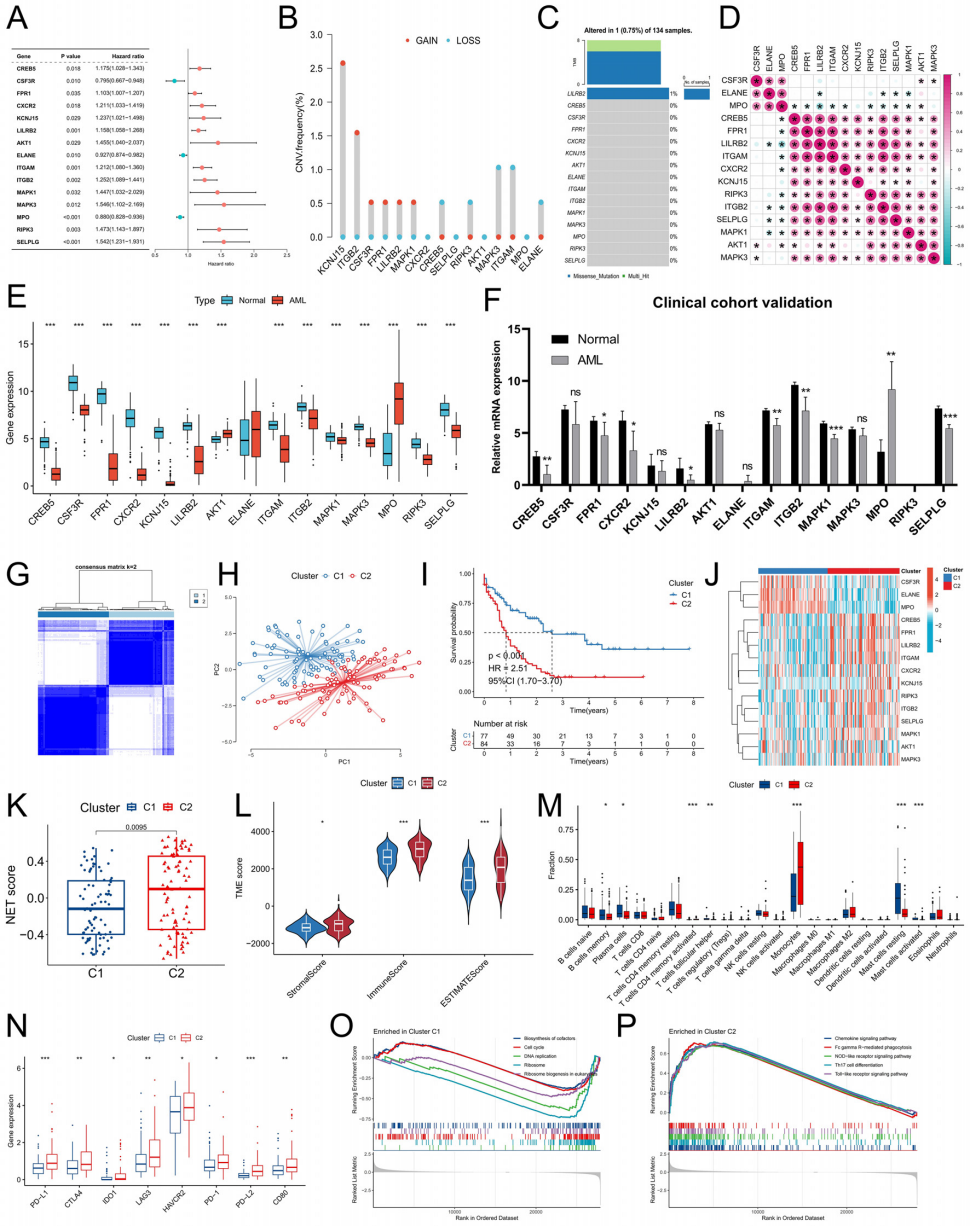
Genetic characteristics of NRG and identification and differential analysis of NET-related molecular subtypes in the TCGA-LAML cohort. **(A)** Univariate Cox regression analysis was conducted to examine the relationship between NRG levels and prognosis in AML patients in the TCGA-LAML cohort. **(B, C)** The frequencies of copy number variations (CNVs) and somatic mutations in the prognostic NRG gene were analyzed in the TCGA-LAML cohort. **(D)** Correlation analysis of prognostic NRG expression. **(E, F)** Variations in prognostic NRG expression between normal samples and AML samples were assessed in both the combined TCGA-LAML cohort and the GTEx-normal cohort **(E)**, as well as the clinical cohort **(F)**. **(G)** The consensus clustering algorithm identified two distinct molecular subtypes. **(H)** The reliability of the clustering was validated via the PCA algorithm. **(I)** Survival analysis revealed significant differences between the two subtypes. **(J)** Differences in the expression characteristics of NRG between the two subtypes were visualized via a heatmap. **(K-N)** Differences in the NET score **(K)**, TME score **(L)**, immune cell infiltration **(M)**, and immune checkpoint expression **(N)** were observed between subtypes. **(O, P)** GSEA identified signaling pathways with significant enrichment differences between molecular subtypes. * P<0.05, ** P<0.01, *** P<0.001. ns, no significance.

We further analyzed the expression characteristics of NRG in AML. Univariate Cox regression analysis revealed a significant correlation between the expression of 15 NRGs and the prognosis of AML patients (P<0.05) (**Figure 2A**). Among these genes, CSF3R, ELANE, and MPO were identified as protective factors for AML patient prognosis, with high expression indicating a better prognosis. Conversely, the remaining 12 NRGs were identified as risk factors for AML and were associated with poor prognosis in patients. Copy number variation analysis of these 15 NRGs revealed a relatively high acquisition frequency for KCNJ15 and ITGB2, whereas MAPK3 and ITGAM presented relatively high copy number loss frequencies (**Figure 2B**). Somatic mutation analysis revealed only one AML sample with a missense mutation in LILRB2 (**Figure 2C**). Expression correlation analysis revealed a significant positive correlation between CSF3R, ELANE, and MPO expression, as well as positive correlations among other NRGs, suggesting potential synergistic effects between these genes (**Figure 2D**). To validate the bioinformatics analysis results, we conducted expression verification for the prognostic NRG identified above. Differential analysis between TCGA-LAML and GTEX-normal samples revealed that, in AML samples, the expression of CREB5, CSF3R, FPR1, CXCR2, KCNJ15, LILRB2, ITGAM, ITGB2, MAPK1, MAPK3, RIPK3 and SELPL was significantly downregulated compared with that in normal samples (**Figure 2E**). Additionally, the expression of AKT1 and MPO was significantly downregulated. Consistent with the public cohort findings (**Figure 2F**), our clinical cohort also exhibited a similar expression trend, which further enhances the reliability of our data analysis.

### Identification of NET-related molecular subtypes in AML

To better assess the molecular characteristics of the NRG in AML patients, we employed a consensus clustering algorithm to categorize them into two subgroups, namely, Cluster C1 and Cluster C2, on the basis of the distinct expression patterns of the 15 prognostic NRGs (**Figure 2G**). The reliability of the molecular subtypes was confirmed via the PCA algorithm (**Figure 2H**). The survival analysis revealed a significantly superior prognosis for patients with the C1 subtype compared to those with the C2 subtype (**Figure 2I**). Interestingly, the C1 subtype presented significantly elevated expression levels of CSF3R, ELANE, and MPO, whereas the remaining NRGs presented higher expression levels in the C2 subtype (**Figure 2J**). Moreover, the C2 subtype displayed higher NET scores as well as immune and stromal scores (**Figures 2K, L**), potentially related to its increased monocyte infiltration proportion (**Figure 2M**). We also observed the upregulation of immune checkpoints such as PD-1, PD-L1, and CTLA4 in the C2 subtype (**Figure 2N**). Pathways associated with cell growth and proliferation, such as the biosynthesis of cofactors, the cell cycle, DNA replication, and ribosome and ribosome biogenesis in eukaryotes, demonstrated increased enrichment scores in the C1 subtype (**Figure 2O**), whereas the activity scores of immune-related signaling pathways, including chemokine signaling pathway, FcγR-mediated phagocytosis, NOD-like receptor signaling pathway and Th17 cell differentiation were higher in the C2 subtype (**Figure 2P**).

### Validating the NET-related molecular subtypes and analyzing the biological differences between the molecular subtypes

**Figure 3:**
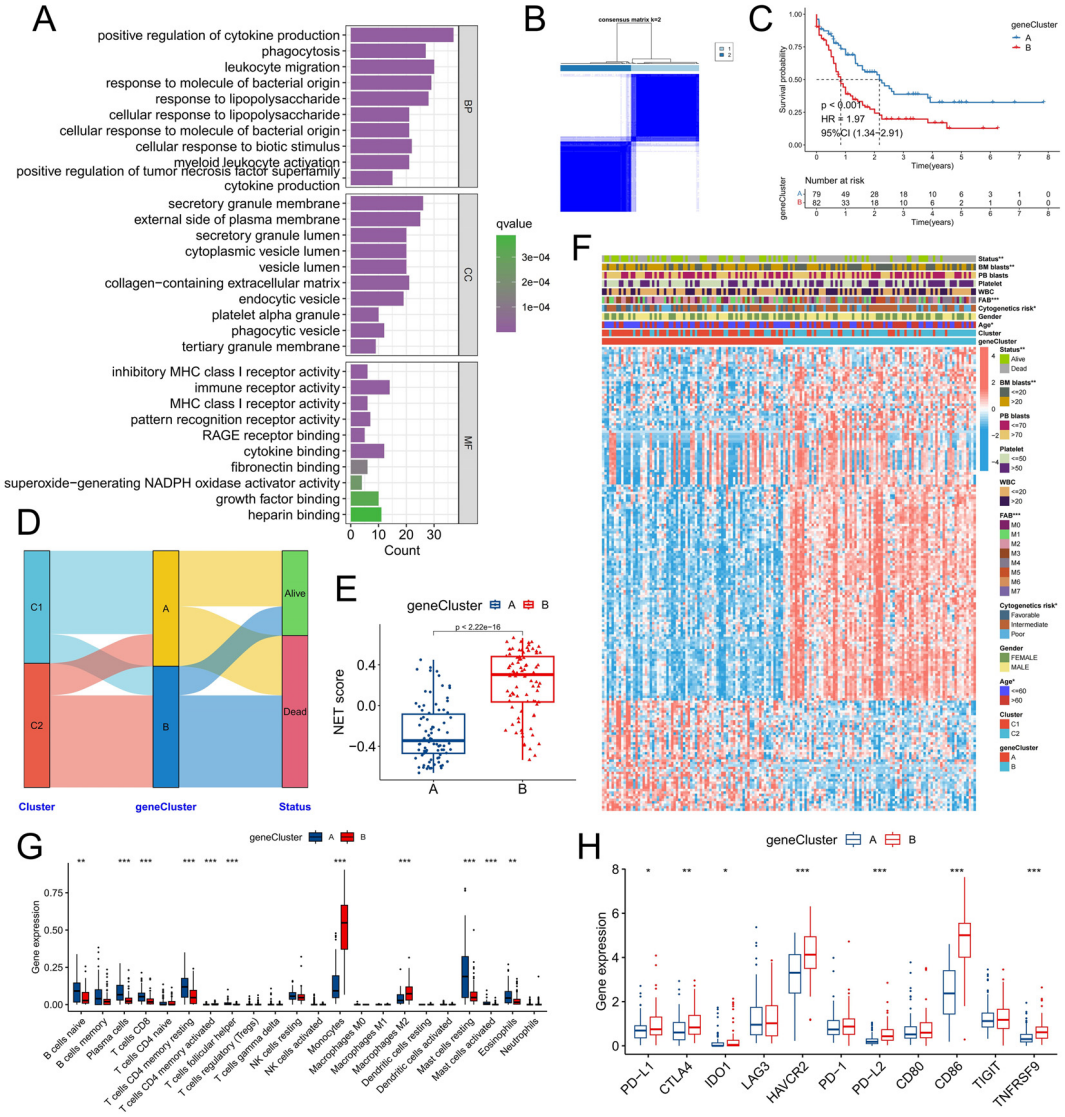
Validation of molecular subtypes in the TCGA-LAML cohort. **(A)** Functional annotation was conducted on genes that were differentially expressed between molecular subtypes. **(B)** Consensus clustering was performed on the basis of the expression of these DEGs. **(C)** Survival analysis was carried out to compare the two gene subtypes. **(D)** The distribution patterns of patients across NRG molecular subtypes and gene subtypes were examined. **(E, F)** Discrepancies in the NET score **(E)**, DEG expression, and clinicopathologic factors **(F)** were observed between the gene subtypes. **(G)** Differences in immune cell infiltration were identified between the gene subtypes. **(H)** Variations in immune checkpoint gene expression were found between the different gene subtypes. * P<0.05, ** P<0.01, *** P<0.001.

To further validate the existence of the two NET-related molecular subtypes, we conducted differential expression analysis of the C1 and C2 subtypes, resulting in the identification of a total of 286 DEGs (**Supplementary Table S2**). The biological functions associated with these DEGs involved signaling pathways such as positive regulation of cytokine production, phagocytosis, and secretory granule membranes, all of which are closely linked to NET release and function (**Figure 3A**). By utilizing the expression profiles of prognosis-related DEGs, we performed consensus cluster analysis and successfully classified patients into two distinct gene clusters: geneClusters A and B (**Figure 3B**). Notably, patients belonging to geneCluster B presented significantly worse outcomes than did those in geneCluster A (**Figure 3C**). Alluvial maps revealed that the majority of C1 subtype patients were predominantly assigned to geneCluster A, whereas most C2 subtype patients were predominantly assigned to geneCluster B; moreover, a greater proportion of deceased patients were present in geneCluster B (**Figure 3D**). Furthermore, the NET score within geneCluster B was significantly higher than that within geneCluster A (**Figure 3E**). This difference was even more pronounced than the disparity between subtypes C1 and C2, suggesting that these two specific genetic subtypes may better discern variations in the tumor microenvironment among individuals with differing levels of NET activity. In terms of clinical characteristics, the C2 subtype encompassed a greater number of elderly patients with adverse cytogenetic risk factors (**Figure 3F**). Immune infiltration analysis revealed that geneCluster A had an increased proportion of naive B cells, plasma cells, CD8+ T cells, resting CD4+ memory T cells, follicular helper T cells, resting mast cells, and eosinophils, whereas monocytes and M2 macrophages constituted a greater fraction within geneCluster B (**Figure 3G**). Additionally, the expression of immune checkpoint genes such as PD-L1, CTLA4, and IDO1 was significantly upregulated in gene Cluster B (**Figure 3H**). Numerous studies have demonstrated that monocytes are involved in chronic inflammation, whereas M2 macrophages hinder the functionality of immune cells through the release of immunosuppressive chemicals. This phenomenon facilitates immune evasion by tumor cells, thereby promoting tumor resistance and progression. Taken together, these findings indicate that there are two distinct NET-related molecular subtypes in patients with AML, which significantly differ in terms of prognosis, clinical characteristics, and the TME.

### Development and prognostic value analysis of the NET-related signature

**Figure 4:**
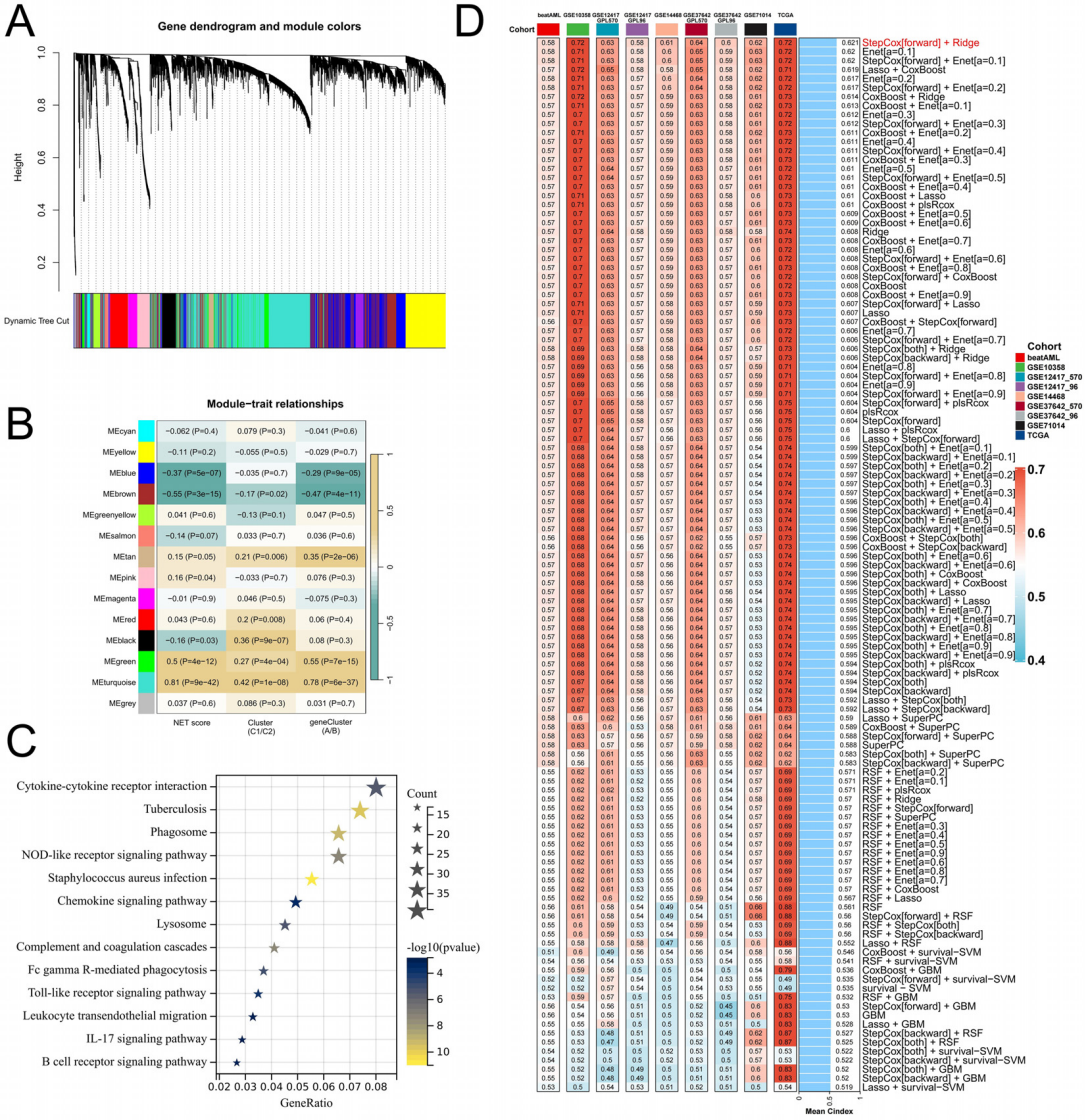
Relevant NRGs were identified via WGCNA and machine learning-based integrators to construct and validate a risk score model. **(A)** Clustering based on different measures (1-TOM) is represented in the dendrogram in the TCGA-LAML cohort. **(B)** Heatmap illustrating the correlations between modules and various phenotypes. **(C)** KEGG enrichment analysis reveals the functional role of genes in the turquoise module. **(D)** A comprehensive computational framework generated a combination of 117 machine learning algorithms, with each model’s C-index calculated across 9 cohorts and sorted on the basis of the average C-index from the validation set evaluation.

**Figure 5:**
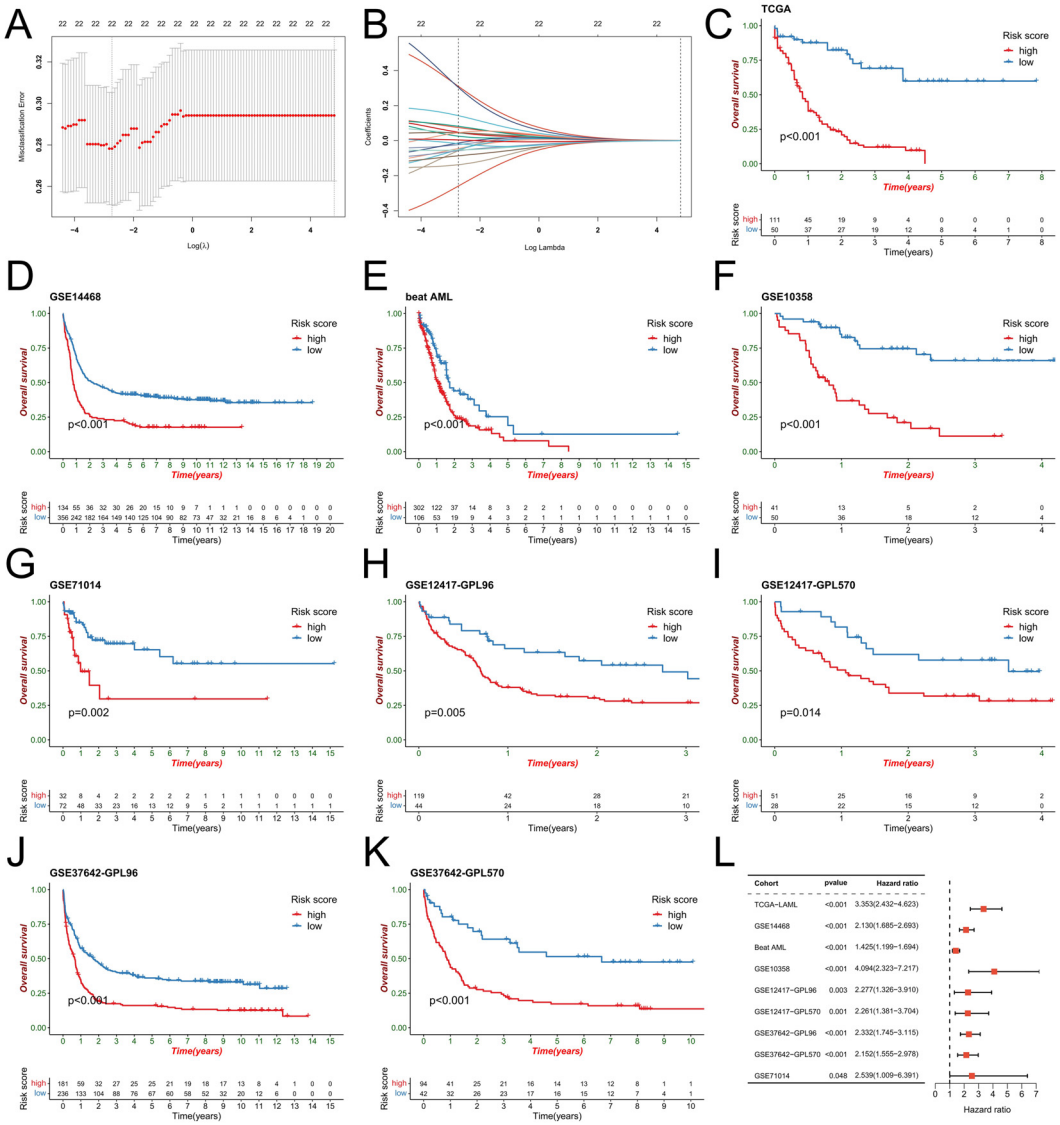
Construction of the risk score model. **(A)** The penalty coefficient was computed for the minimum 10-fold cross-validation error point to identify the corresponding model gene. **(B)** Estimation of the coefficients for the model genes. Each line corresponds to a model gene. **(C-K)** Utilizing an optimal cutoff value, patients in the AML cohort were stratified into high- and low-risk score groups for survival analysis. **(L)** Univariate Cox regression analysis demonstrated the predictive ability of the risk score across 9 AML cohorts.

**Figure 6:**
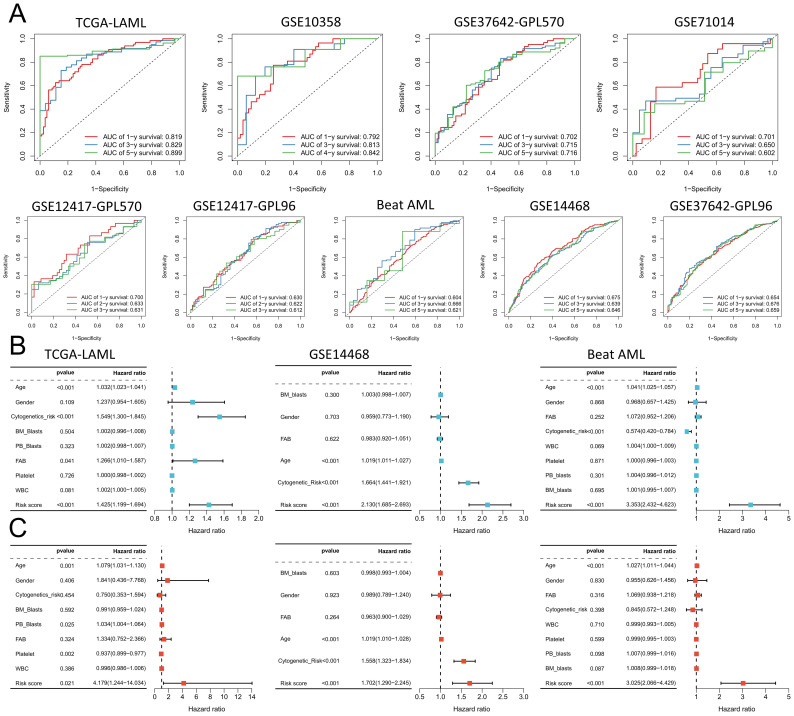
Validation of the prognostic predictions of the risk score models. **(A)** The risk scores of the 9 cohorts were analyzed via receiver operating characteristic (ROC) curve analysis. **(B, C)** The TCGA-LAML, GSE14468, and Beat AML cohorts were subjected to univariate **(B)** and multivariate **(C)** Cox regression analyses of clinicopathologic factors and risk scores.

To better elucidate potential NRGs and biological pathways, we employed the WGCNA algorithm to identify coexpressed gene modules associated with NET scores, Cluster C1/C2, and geneClusters A/B (**Figure 4A**). The turquoise module exhibited the strongest correlation with all three phenotypes (NET score: R=0.81, P=9e-42; Cluster C1/C2: R=0.42, P=1e-08; geneCluster A/B: R=0.78, P=6e-37), thus warranting our focus on this module (**Figure 4B**). This module encompasses 1385 genes that are enriched primarily in immune-related pathways such as the cytokine−cytokine receptor interaction, tuberculosis, phagosome, and NOD-like receptor signaling pathways (**Figure 4C**). We then utilized these genes to construct NET-related signatures and elucidate their prognostic value and therapeutic value. Univariate Cox regression analysis was employed to identify 22 prognostic genes, followed by the construction of prognostic risk score models on the basis of 117 combinations of 10 machine learning algorithms. The predictive power of all the models was assessed by calculating the C-index for each model in all the cohorts. Among the validation cohorts, we selected the model combination with the highest average C-index, an algorithm composed of StepCox (forward) and Ridge (**Figure 4D**). The StepCox algorithm identified the most valuable genes, whereas the ridge algorithm further constructed a highly valuable model consisting of 22 genes (**Figures 5A, B**). We subsequently calculated the risk score for each sample across all cohorts. Patients with high risk scores exhibited poor clinical outcomes in all nine AML cohorts examined (**Figures 5C–K**). Further univariate Cox regression analysis confirmed a significant correlation between the risk score model and prognosis among AML patients (P<0.05) (**Figure 5L**). ROC curve analysis revealed that in the TCGA-LAML cohort, the area under the curve (AUC) values for predicting 1-year, 3-year, and 5-year survival rates among AML patients were 0.819, 0.829, and 0.899, respectively; similar results were observed in other cohorts where the AUC values exceeded 0.6, thus fully validating the accuracy of our risk score model’s prognostic predictions (**Figure 6A**). The three AML cohorts, TCGA-LAML, GSE14468, and Beat AML, contained additional clinical information that allowed us to perform independent prognostic analyses, revealing significant independent prognostic value associated with our risk score model through both univariate and multivariate Cox analyses (P<0.05) (**Figures 6B, C**).

### Comparison of prognostic signatures in AML

**Figure 7:**
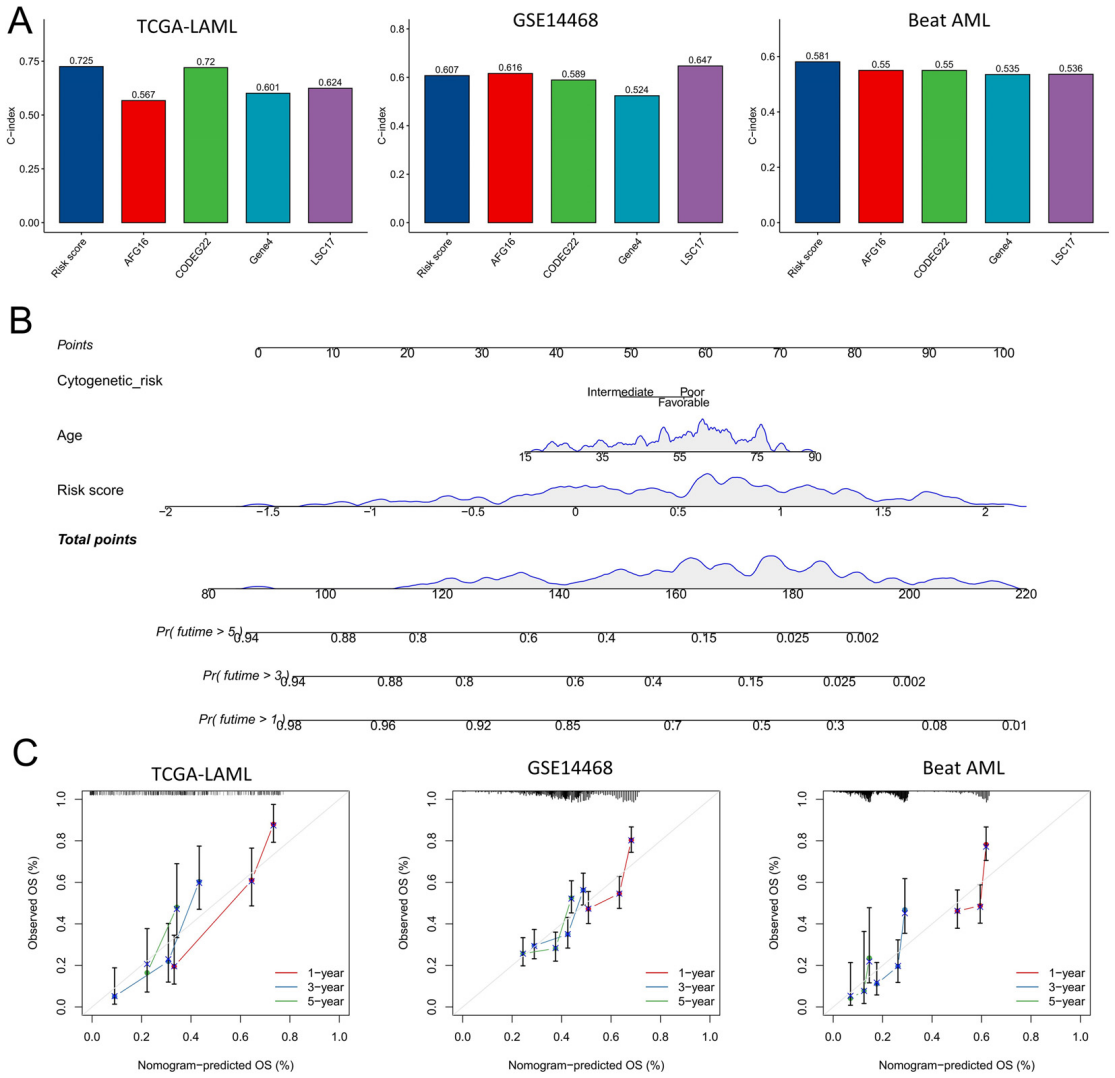
Model comparison and construction of the nomogram. **(A)** The performance of the risk score model was evaluated against the C index of previously published signatures in the TCGA-LAML, GSE14468, and Beat AML cohorts. **(B)** A nomogram incorporating both clinicopathological factors and the risk score was developed to predict overall survival (OS) in patients with AML. The following three scales indicate the likelihood of survival exceeding the respective time periods. **(C)** A calibration curve was used to assess the precision of the nomogram for predicting OS.

Next, we integrated the risk scoring model with gene expression analyses derived from large-scale *in vitro* and *in vivo* CRISPR-Cas9 knockout screening (AFG16) (25), stem cell subpopulation-defined phenotypes (LSC17) (26), multiple paired comparisons between AML subgroups and healthy controls (CODEG22) (27), or capturing intratumoral heterogeneity in AML (GENE4) (28) to generate a comprehensive comparison of robust prognostic signatures. In the three cohorts with extensive clinical annotations, namely, TCGA, GSE14468, and Beat AML, the risk scores exhibited a slightly higher C-index than did the other signatures on the basis of gene expression analysis (**Figure 7A**). However, their values were lower than those obtained from AFG16 and LSC17 within the GSE14468 cohort. Overall, these findings demonstrate that risk scores are independent of previously published signatures relying on gene expression analysis and can serve as reliable prognostic predictors for patients with AML. Finally, we incorporated clinicopathological factors significantly associated with AML prognosis identified through univariate Cox analysis into a nomogram for predicting overall survival (OS) in AML patients (**Figure 7B**). The calibration curves of the TCGA, GSE14468, and Beat AML datasets confirmed the accuracy of our nomogram predictions (**Figure 7C**).

### Analysis of variations in chemotherapy sensitivity and immunotherapy response between risk score groups

**Figure 8:**
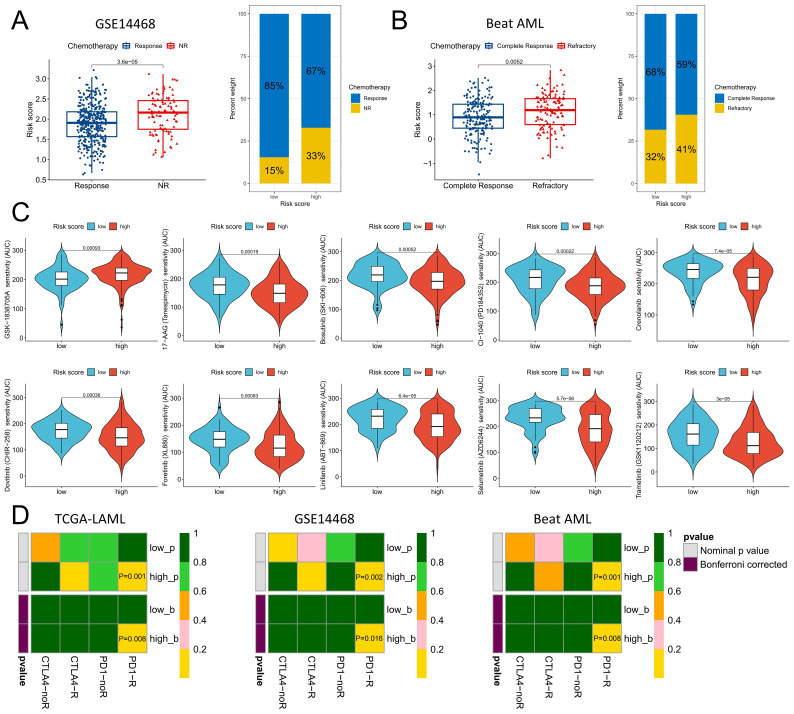
Distinguishing the responsiveness to chemotherapy and immunotherapy between the high- and low-risk score groups. **(A)** Variations in risk scores were observed among patients who exhibited a positive response to chemotherapy compared with those who did not, within the GSE14468 cohort. **(B)** Distinctions in risk scores were identified between patients responsive or resistant to chemotherapy within the Beat AML cohort. **(C)** Chemotherapeutic agents displaying varying degrees of sensitivity across different risk score groups within the Beat AML cohort. **(D)** Anticipating the efficacy of anti-PD-1 and anti-CTAL4 immunotherapy on the basis of diverse risk score categories across the TCGA-LAML, GSE14468, and Beat AML cohorts. The P-value indicates the potential efficacy of the corresponding immunotherapy; a smaller P-value suggests a higher level of benefit.

To comprehensively evaluate the role of risk scores in chemotherapy and immunotherapy for AML, we conducted a systematic analysis. Compared with responders, patients who did not respond to chemotherapy in the GSE14468 and Beat AML datasets presented significantly elevated risk scores, with a greater proportion of patients in the high-risk group failing to respond to chemotherapy (**Figures 8A, B**). The Beat AML cohort included drug sensitivity data, specifically for isolated AML cells. On the basis of a significance level of P<0.001, we identified a set of drugs exhibiting significant sensitivity differences between the high- and low-risk groups, with the low-risk group displaying greater sensitivity toward GSK-1838705A. In the high-risk group, 17-AAG (tanespimycin), bosutinib (SKI-606), CI-1040 (PD184352), crenolanib, dovitinib (CHER-258), foretinib (XL880), linifanib (ABT-869), selumetinib (AZD6244) and trametinib (GSK1120212) were more sensitive (**Figure 8C**). With respect to the prediction of the immunotherapy response, all three cohorts indicated that the high-risk group exhibited an improved therapeutic response to PD-1 therapy after correction for multiple testing at p<0.05 (**Figure 8D**).

## Discussion

AML patients with aberrant myeloid hematopoiesis, particularly disordered neutrophil differentiation, exhibit impaired immune function and frequently experience complications such as severe infections (29). NETs play a crucial role in combating infections (13). Previous studies have demonstrated that the formation and release of NETs are compromised in AML (10), potentially serving as a significant mechanism contributing to the unfavorable prognosis observed in AML patients. This study aimed to analyze the comprehensive expression profile of NRG to elucidate the underlying mechanism of NETs in AML.

The NET score was initially quantified to assess the activity of NETs, which was further validated via the GSVA algorithm and correlation analysis. AML patients presented a significant reduction in NET scores, which correlated with the downregulation of overall NRG gene expression. However, a high NET score in AML patients indicates increased activity of the cancer signature pathway and predicts poor prognosis. This was accompanied by reduced infiltration of immune effector cells and an increased ratio of monocytes to neutrophils. Additionally, there was a positive correlation between the NET score and immune checkpoint expression. Subsequent cluster analyses confirmed distinct molecular subtypes with significantly different NET scores in AML patients. Notably, the low-NET score subtypes (Cluster C1 and geneCluster A) presented increased proportions of CD4+ and CD8+ T cells as well as B cells, whereas the high-NET score subtypes (Cluster C2 and geneCluster B) presented increased numbers of monocytes and M2 macrophages along with elevated levels of immune checkpoints such as PD-1, PD-L1, and CTLA4. On the basis of these findings, it can be speculated that a high NET score has pronounced immunosuppressive effects, whereas increased infiltration of mononuclear macrophages is associated with chronic inflammation (30). Moreover, M2 macrophages play a crucial role in chronic inflammation by inhibiting tumor immune cell activity through the secretion of various cytokines and surface molecules, leading to immune evasion (31). Chronic inflammation has also been implicated as an additional factor contributing to the development of AML (32). In light of these adverse factors, immunity is compromised in AML patients, resulting in reduced anti-infection capabilities. Although a higher NET score may indicate attempts by neutrophils to increase the body’s anti-infection ability through improved formation and release of NETs, this seems ineffective in preventing infection. Of course, these bioinformatics findings presented herein suggest several potential phenomena and biological associations, and further experimental validation is required to substantiate these observations. Furthermore, our observations indicate that patients with the low-NET score subtype, who exhibited a more favorable prognosis, demonstrated heightened activity in cell cycle and DNA replication pathways. Typically, AML treatment predominantly relies on chemotherapy, particularly drugs targeting the cell cycle. Cancer cells with elevated cell cycle and DNA replication activities are more susceptible to these agents, thereby enhancing the efficacy of chemotherapy. Moreover, highly active cancer cells depend on continuous proliferation signals; inhibiting these signals can induce cell death. Active DNA replication can also result in genomic instability, which, although it may potentially promote cancer progression, can also render cancer cells more vulnerable to defects in DNA damage repair mechanisms, increasing their sensitivity to therapeutic interventions. Additionally, rapidly proliferating cancer cells may express a greater number of neoantigens, making them more readily recognized and targeted by the immune system, thus augmenting the effectiveness of immunotherapy.

Second, we conducted WGNCA analysis and identified a set of genes that exhibited significant associations with the NET score and molecular subtypes. These genes are enriched in pathways such as the cytokine−cytokine receptor interaction, tuberculosis, phagosome, and NOD−like receptor signaling pathways, which are closely related to NET regulation (33). We subsequently comprehensively analyzed the prognostic value of these potentially related NRGs. Machine learning algorithms have been employed for analyzing multiple types of omics data effectively (34). In this study, we included 9 multicenter cohorts of AML patients and selected the best combination of machine learning algorithms out of 117 options to overcome algorithm selection limitations. To prevent overfitting during the construction of the risk score model, that is, good predictive performance on training data can be generalized to other validation datasets, we used the average C-index from multiple validation datasets as our selection criterion. Then, StepCox (forward) and Ridge were utilized for training purposes. The risk score model demonstrated excellent performance on both the training and validation datasets. In all 9 AML cohorts examined, patients with high risk scores had a significantly worse prognosis. Univariate and multivariate Cox regression analyses confirmed that the risk score model was an independent predictor of AML prognosis. This finding was further supported by ROC curve analysis, which validated its prognostic value. The nomogram constructed by integrating the risk score model with age and cytogenetic risk demonstrated remarkable accuracy in predicting the OS of patients with AML. Consequently, our study presents an independent and reliable prognostic assessment tool for evaluating AML prognosis.

Finally, our analysis revealed that patients with refractory or chemotherapy-unresponsive AML presented significantly elevated risk scores, indicating the potential of the risk score model in predicting chemotherapy sensitivity among AML patients. By utilizing data on drug treatment in ex vivo AML cells from patients, we identified a cluster of drugs displaying increased sensitivity within the high-risk score group, thereby providing valuable insights into treatment strategies for these individuals. Furthermore, patients with a high risk score were also predicted to be responsive to anti-PD-1 therapy, suggesting that those with an elevated risk score may derive greater benefits from immunotherapy. Our findings underscore the importance of the risk score model as a valuable biomarker for guiding precision therapy in AML patients and potentially improving patient outcomes while reducing unnecessary treatment costs. Overall, this risk score model holds promise as a clinical tool enabling physicians to make personalized treatment decisions. However, it is important to acknowledge certain limitations of this study. For example, although we conducted analyses and validated NET-related signatures across nine AML cohorts, further confirmation through larger-scale multicenter real-world cohorts is still warranted. Additionally, more extensive *in vitro* and *in vivo* experiments are needed to elucidate the biological function of NRG in relation to AML. Moreover, despite our predictions regarding the sensitivity of different risk score subgroups to small-molecule agents and immunotherapy interventions, validation through *in vitro* drug assays and clinical trials is necessary.

## Conclusion

The present study comprehensively analyzed the molecular characteristics of NETs in AML and elucidated their associations with the TME and prognosis in AML patients, thereby providing novel insights into the molecular mechanisms underlying AML progression. NET-related signatures, which are constructed via diverse machine learning algorithms, hold great promise as valuable tools for prognostic prediction, prevention, and personalized medicine in AML patients.

## Data Availability

The data presented in the study are deposited in the GEO repository, accession number GSE10358, GSE12417, GSE37642, GSE71014, GSE14688.
